# Interorgan Molecular Communication Strategies of “Local” and “Systemic” Innate Immune Responses in Mosquito *Anopheles stephensi*

**DOI:** 10.3389/fimmu.2018.00148

**Published:** 2018-02-20

**Authors:** Tanwee Das De, Punita Sharma, Tina Thomas, Deepak Singla, Sanjay Tevatiya, Seena Kumari, Charu Chauhan, Jyoti Rani, Vartika Srivastava, Ramandeep Kaur, Kailash C. Pandey, Rajnikant Dixit

**Affiliations:** ^1^Host-Parasite Interaction Biology Group, ICMR-National Institute of Malaria Research, New Delhi, India; ^2^Department of Biotechnology, Delhi Technological University, Shahbad Daulatpur, New Delhi, India; ^3^Department of Biochemistry, National Institute for Research in Environmental Health, Indian Council of Medical Research, Bhopal, India

**Keywords:** mosquito, innate immunity, antimicrobial peptide, fat body, midgut, hemocyte

## Abstract

Mosquitoes that transmit many deadly infectious diseases also need to keep fighting against many microbial infections. Constitutive expression of multiple antimicrobial peptides (AMPs) in almost all body tissues is believed to facilitate the effective management of these local infections. When any infection breaches the local barrier, AMPs are induced rapidly in non-target tissues such as hemocytes (HCs) and establish their co-ordination with systemic immune effectors to clear off the body infection. But how interorgan immune communication is managed during local and systemic infections remain largely unknown. To understand this interorgan molecular relationship, we identified, extensively profiled and compared the expression of AMPs in three important mosquito tissues *viz*. midgut, fat body (FB), and HCs. *dsRNA*-mediated AMPs silencing suggests that mosquito tissues are able to manage an optimal expression of AMPs at the physiological level. We also examined the possible contribution of two important immune regulator genes relish (REL) and nitric oxide synthase, controlling AMPs expression in these tissues during local or systemic infections. We show that each tissue has a unique ability to respond to local/systemic challenges, but HCs are more specialized to recognize and discriminate-specific antigens than gut and FB. Our investigation also revealed that both REL and NO participate in the overall management of the interorgan immune responses, but at the same time each tissue also has its own ability to maintain the interorgan flow of signals. In our knowledge, this is the first large-scale study examining the interorgan immune relationship in the mosquito.

## Introduction

Vector-borne diseases not only cause huge morbidity and mortality but also have an impact on the economic growth. In the era of genomics, now entomologists are taking new ways of “in-depth” understanding of the mosquito biology. Ongoing genome editing laboratory experiments strongly support the idea that target-specific genetic modification could enable us to alter and/or suppress natural vector population (1). One of the key idea includes blocking the parasite/virus development within the mosquito host, and hence the transmission of disease (2). However, bringing such scientific concepts in operation requires a deep knowledge of molecular interactions linked to vector’s defense mechanisms and pathogen’s ability to sustain within the hostile environment of the mosquito host.

A vast majority of literature clearly demonstrates that insects are evolved with a well-defined molecular architecture of innate immune components, which not only control “*Local*” (first-line defense) and “*Systemic*” (second-line defense) infections but also maintains tissue specificity and physiological integrity (3). When any microbial pathogens breach the “*local*” barriers, e.g., cuticle, trachea, midgut (MG) etc., a “*systemic*” response gets activated in the fat body (FB) or in the hemocytes (HCs) to clear off the infection remnants (4). Similarly, FB is also one of the principal tissue sites for the production and secretion of immune molecules, especially antimicrobial peptides (AMPs) which are rapidly induced in response to any exogenous exposure (5, 6).

Mosquito HCs encode diverse nature of molecular factors which not only contribute to maintaining physiological homeostasis but also regulate many cellular and humoral innate immune responses including phagocytosis, coagulation, and melanization (7–9). Each organ is specialized to perform their respective functions, but a great deal of interorgan communication and adjustment of immune regulators is essential to balance and maintain homeostasis during any altered pathophysiological condition. The mechanism for this co-ordination of interorgan immune network during any “local” or “systemic” infection is not well known.

In *Drosophila*, several recent studies provide evidence of molecular communication between different immune tissues (10–13), however, such evidence are lacking in mosquitoes. A recent study by Ramirez et al. (14) indicates that oral supplement of bacteria significantly alters AMPs expression in the MG as well as the FB of the mosquito *Aedes aegypti*. Studies in *Anopheles* mosquitoes also document that induction of MG associated nitric oxide (NO) kills parasites (15–23). A systemic bacterial immune challenge caused upregulation of nitric oxide synthase (NOS) in the hemocoel, indicating that free radical NO is an important modulator of antimicrobial immune response in the mosquito *An. gambiae* (24). In fact, MG of the blood feeding insects/mosquitoes provides unique sites for multi-taxon interactions including gut flora, pathogens, vertebrate blood factors, etc. This may not only affect the vector physiology but also significantly influence immune responses (14, 25). Additionally, rapid blood meal digestion and gut microbe-pathogen interaction significantly alters the level of redox molecules such as NO, hydrogen peroxide, superoxide, which modulate immunosignaling to manage the infection and repair of the damaged cells (26–28).

Though it is clear that rapid induction of harmful NO or reactive oxygen species kills the pathogen (29), but how the AMPs expression is regulated during acute local and/or systemic infections remains unknown. In *Drosophila* both immunodeficiency (IMD)/TOLL pathways control transcriptional activation of AMPs, where toll pathway counteracts fungal and Gram-positive bacterial infections through nuclear translocation of *Dif* and IMD pathway deals with bacterial infection *via* nuclear translocation of Relish (REL) (30–36). In the absence of *Drosophila Dif* homolog, mosquito immune gene transcription is dominantly regulated by *Rel1* and *Rel2* transcription factors (37). We hypothesized that each tissue must have the ability to sense, communicate, and guide the flow of signals to regulate the AMPs expression. Thus, in the present investigation we attempted to clarify that (i) whether tissue-specific AMPs expression alters in response to microbial challenge; (ii) do FB and HC work synergistically or independently, specifically during systemic immune challenge; (iii) how AMPs expression alters in the HCs or FB during blood meal digestion and gut flora proliferation in the MG; and (iv) whether transcription factor *Rel* and NO signaling molecule also contribute in the management of interorgan immune signals.

Initially, we predicted, identified, and cataloged putative AMPs from the available genome of the mosquito *An. stephensi*, an urban malaria vector in India (38). Next, we profiled and examined the relative expression of selected family genes of AMPs in multiple organs. Through dsRNA silencing, we examined how different tissues manage their co-ordination in the absence of any one of the AMP family member protein. Later, we monitored the influence of natural gut flora on the AMPs expression during blood meal digestion. Lastly, we also examined the possible contribution of *Rel*- and *NOS*-mediated immune regulation of AMPs expression in different immune tissues. Our data strongly suggests that MG flora significantly alters the local response of AMPs during blood meal digestion. Exogenous/endogenous microbial exposure influence local and systemic responses, but distinct AMPs manage immune response in the different tissues. Our functional genomics analysis of AMPs network co-ordination and comparative profiling of their immune regulators provides initial evidence that each tissue has a synergistic ability to manage “local” and “systemic” infections. Any further understanding of the factors controlling interorgan immune communication could enable us to translate this knowledge to design new molecular weapons to block pathogen development and its transmission by vector’s genetic modification strategies.

## Experimental Design and Methodology

**Figure 1:**
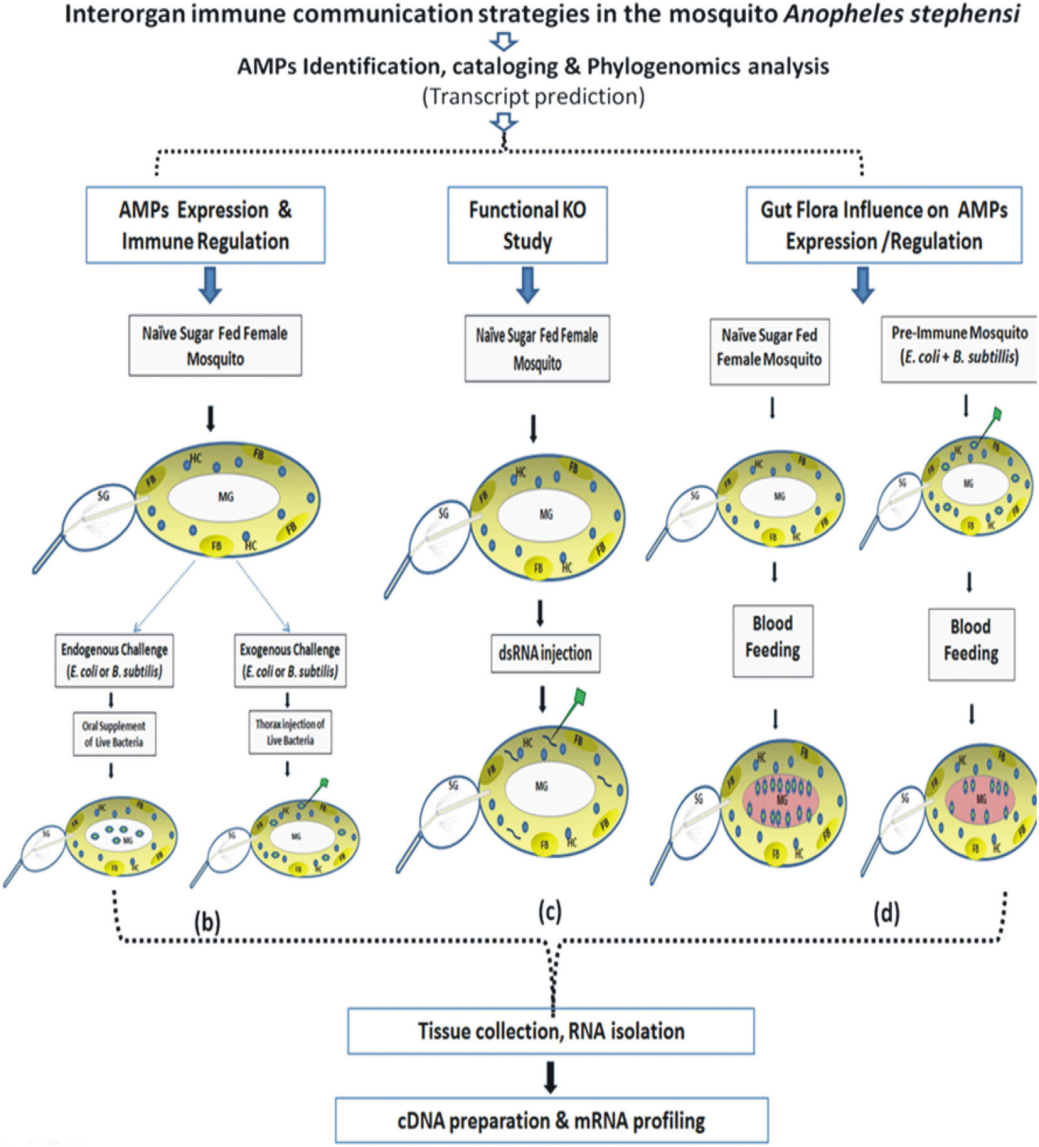
Technical overview and basic methodology followed to understand the molecular and functional correlation of interorgan immune network communication. Fat body (FB): yellow color; hemocyte (HC): blue circle; midgut (MG); salivary gland (SG); bacteria: green colored stars (see text for detail).

The technical design and experimental workflow are shown in Figure 1.

(a) *AMP identification, cataloging, and phylogenomics analysis*: A reverse BLAST approach was applied against *An. stephensi* genome database, by querying the putative AMPs transcripts database originating from *An. gambiae, A. aegypti, Culex quiquifaciatus*, and *D. melanogaste*r (http://cegg.unige.ch/Insecta/immunodb). We also downloaded the complete transcripts from *An. stephensi* (*SDA-500 and Indian*) from the vectorbase database (www.vectorbase.org). Reference AMPs were used as BlastN query with *An. stephensi* transcripts as input database at e-value 1e^−03^. All the blast hits were filtered out for query coverage ≥ 40%. The hits obtained were assigned the corresponding class and used for multiple sequence alignment using muscle software. Finally, the alignment file from each family of genes was used as input for PhyML tool for the phylogenetic tree using default parameters.
(b) *AMPs expression and immune regulation*: This part depicts schematic overview of 3–4 days old adult female naïve mosquitoes that were given live microbial challenge either by oral supplementation (endogenous exposure) eliciting dominant AMPs response in the MG epithelium (spiked blue/green dotes representing bacteria in MG); or by thorax injection (exogenous exposure) expected a direct interaction of bacteria (spiked blue/green dotes) with HCs (round blue cells/HC) or FB (oval light yellow shape/FB).
(c) *Functional KO study*: This part depicts dsRNA-mediated gene silencing strategy to deplete the native mRNA population by injecting purified dsRNA (small rod shape) in the thorax of the adult female mosquitoes.
(d) *Gut flora influence on AMPs expression and regulation*: A schematic overview to examine AMPs expression in response to blood meal (pinkish-red color in MG) induced gut flora expansion (spiked blue/green dotes representing bacteria in naïve mosquito) and to test the hypothesis whether pre-immunization, i.e., exogenous challenge of mixed bacterial population in the thorax alters the proliferating population of gut bacteria in blood-fed mosquitoes.

### Mosquito Rearing

A cyclic colony of the mosquito *An. stephensi* was maintained at 28 ± 2°C and relative humidity of 80% in the insectary, at NIMR. All protocols for rearing, maintenance of the mosquito culture were approved by ethical committee of the institute. Live animal (rabbit) blood was fed for mosquito cycle maintenance and experimental purpose.

### Antibiotic Treatment

Prior to blood feeding, freshly emerged 50 adult mosquitoes were fed on sterile water supplemented with antibiotic cocktail (see below) for 3–4 days to remove the MG flora. An equal number of untreated mosquitoes were also grown in identical experimental conditions except for them water was supplemented without the antibiotic. All experiments were performed in a way to minimize the risk of contamination. The removal of bacterial gut flora was examined by LB plate assay. In this assay, the MG from control and antibiotic-treated adult female mosquitoes were dissected and homogenized in sterile phosphate buffer saline (PBS), followed by plating the crude diluted homogenate (1:10 or 1:50) on LB plates which were then incubated overnight at 37°C for the growth of LB cultivable microbial flora. After multiple experimental trials and different antibiotic combinations, we finally observed a maximum removal of the flora by the treatment of penicillin, amoxicillin, and gentamycin (~100 μg/ml each) combination (Figure S1 in Supplementary Material). Additionally, the depletion of the gut flora was also verified through 16sRNA based real-time (RT) PCR analysis assay and adopted for subsequent analysis of AMPs.

### Immune Challenge

*Escherichia coli* (EC) and *Bacillus subtilis* (BS) were grown overnight in LB medium, precipitated, washed and re-suspended in PBS. A bacterial suspension of 100 nl (EC at O.D. 600 = 0.59 and BS at O.D. 600 = 0.52) was injected into the thorax of cold anesthetized mosquito using nano injector. 100 nl of sterile PBS was injected into the control mosquitoes. The same bacterial suspension was used for bacterial feeding and a suspension of bacterial culture of O.D. = 1 was prepared in 5% sterile sugar solution. Mosquitoes were fed with the respective live bacterial solution using a cotton swab. Control mosquitoes were fed on 5% sterile sugar solution only. Approximately a total of 135–150 adult female mosquitoes were kept for control or immune challenge for at least three minimum experimental replicates. We monitored the survival rate of mosquitoes after immune challenge and observed 100% survival in the case of an endogenous challenge, however, only 50–60% survival was observed in the exogenous challenge.

### RNA Isolation and cDNA Synthesis

Total RNA was isolated from MG, FB, HC tissues of mosquito and different developmental stages using the standard Trizol (Invitrogen) method. A minimum of 20 mosquitoes was dissected to pool and collect MG or FB tissue, while HC was pooled from at least 40 mosquitoes, for each experimental set of RNA isolation. Flushing method opted for HC collection as described previously (39). Briefly 2–3 µl of Schneider’s (RPMI):FBS:citrate buffer (60:10:30) were injected into the lateral wall of mesothorax of cold anesthetized mosquitoes, followed by flushing out the diluted hemolymph with additional 3–5 µl of Schneider’s (RPMI), by clipping off the last abdominal segment and the diluted hemolymph was collected in Trizol. First-strand cDNA was synthesized using a mixture of oligo-dT and random hexamer primers and Superscript II reverse transcriptase (Verso cDNA synthesis Kit, Cat#AB-1453/A, EU, Lithuania).

### RT-PCR and Relative Gene Expression Analysis

For differential expression analysis, routine RT-PCR and agarose gel electrophoresis protocols were used. Following PCR amplification parameters were used: 95°C/5 min (1 cycle); 95°C/30 s, 52°C/30 s, and 72°C/30 s (32 Cycles); and final extension was performed at 72°C/5 min. The relative gene expression was assessed by SYBR green qPCR (Thermo Scientific) in Illumina Eco RT-PCR machine. PCR cycle parameters involved an initial denaturation at 95°C for 15 min, 40 cycles of 10 s at 95°C, 15 s at 52°C, and 22 s at 72°C. Fluorescence readings were taken at 72°C after each cycle. The final steps of PCR at 95°C for 15 s followed by 55°C for 15 s and again 95°C for 15 s was completed before deriving a melting curve. Each experiment was performed in three independent biological replicates, except duplicate for HC-related experiments due to RNA limitation from the HCs. The relative quantification results were normalized with internal control Actin gene and analyzed by 2^–ΔΔCt^ method (40). For a comprehensive understanding, data were interpreted to evaluate a general response, however, where ever required “test” sample data was compared with “control” data set and statistically analyzed using Student’s *t*-test. The primers used for each gene are shown in Table S3 in Supplementary Material.

### dsRNA-Mediated Gene Silencing

For gene silencing of cecropin family (C1, C2, and C3), we used RT-PCR amplification strategy with newly designed primers carrying T7 overhang sequence: dsrCec1: forward 5′-TAATACGACTCACTATAGGGTGTCAAGGCTCTTGGATGAA-3′ and reverse 5′-TAATACGACTCACTATAGGGTGACAGCGGTTTGATTAGAGG-3′. Cec2: forward 5′-TAATACGACTCACTATAGGG CTGGTGCTGATGGCTGTCT-3′ and reverse 5′-TAATACGACTCACTATAGGG GCGCTTTATTGGAACTGCAT-3′. Cec3: forward 5′-TAATACGACTCACTATAGGGTCCCTTTCTGTATCCGCCTA-3′ and reverse 5′-TAATACGACTCACTATAGGGTCAGGTCCGCTCCATTTATC-3′. The amplified PCR product was examined by agarose gel electrophoresis, purified, quantified, and subjected (~1 ug) to double-stranded RNA synthesis using Transcript Aid T7 high-yield transcription kit (Cat# K044, Ambion, USA). To generate control dsRNA, we use LacZ gene of EC similar to our test gene. About 69 nl (3 µg/ul) of the corresponding dsRNA in nuclease-free water was injected into the thorax of cold anesthesized 1–2-day old female mosquito using nano- injector (Drummond Scientific, CA, USA). The silencing of the respective gene was confirmed by quantitative RT-PCR 3-day post-dsRNA injection.

### ROS Determination of Mosquito MG during Endogenous Bacterial Challenge

To determine the level of ROS generation in the MGs during endogenous bacterial challenge (24 h), we incubated the MG of naïve and bacterial challenged mosquitoes with a 2 mM solution of the oxidant-sensitive fluorophores, CM-H2DCFDA [5-(and-6)-chloromethyl-29,79-dichloro-dihydrofluorescein diacetate, acetyl ester] (Sigma). After a 20-min incubation at room temperature in the dark, the MGs were washed three times with PBS. Next, the MGs were transferred to a glass slide in a drop of PBS and checked the fluorescence intensity under a fluorescent microscope at 490 nm.

## Results

### AMP Identification, Cataloging, and Phylogenomics Analysis

**Figure 2:**
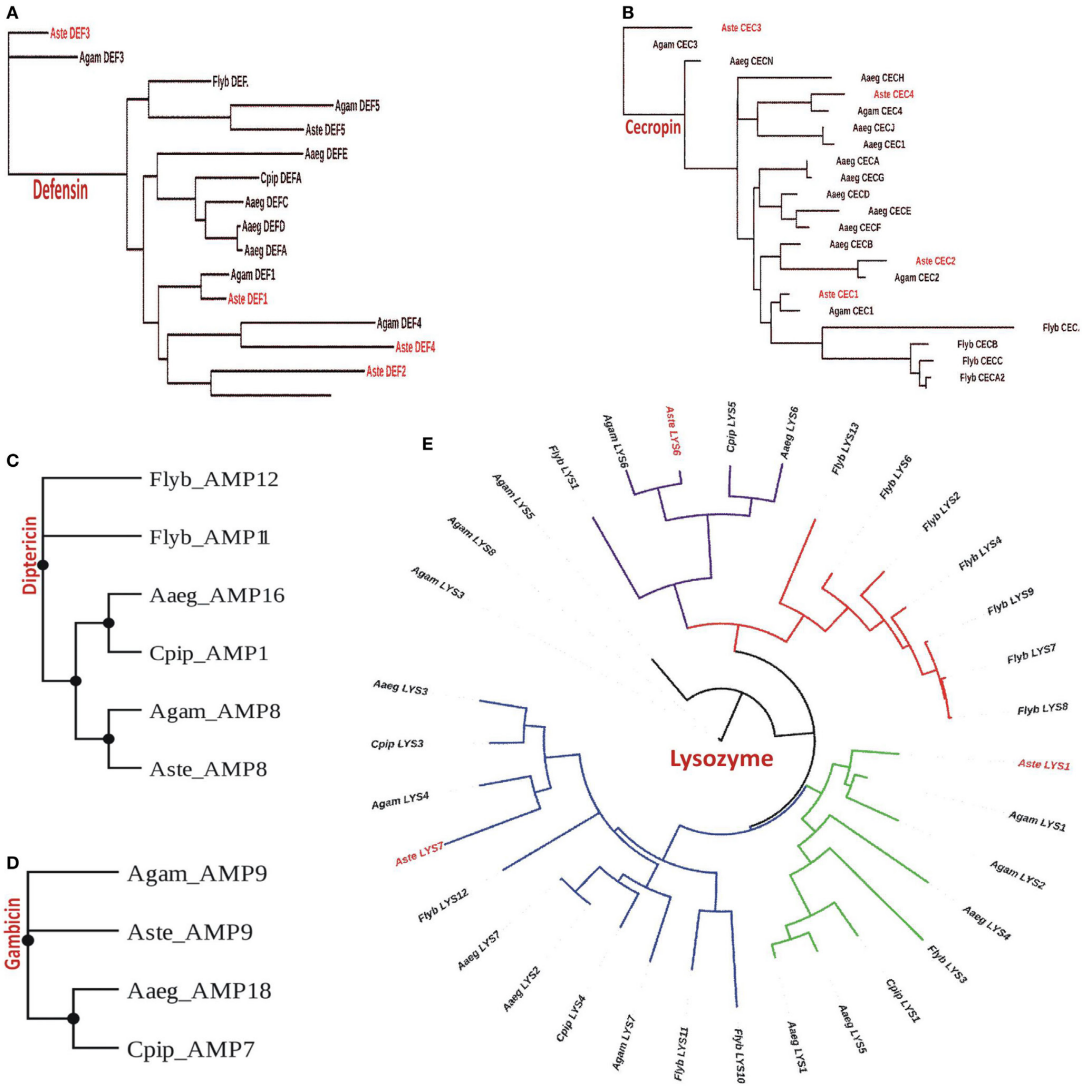
Phylogenomic analysis of selected antimicrobial peptides (AMPs): **(A)** defensin; **(B)** cecropin; **(C)** diptericin; **(D)** gambicin; and **(E)** lysozyme.

Our analysis suggested that *An. stephensi* genome predicted transcript database which includes both SDA-500 Pakistani strain and Indian strain, has a total of 11 AMPs genes of which 4 belongs to cecropin and 5 to defensin family (Table S1 in Supplementary Material). However, only three genes from each cecropin and defensin families were observed from Indian strain. We also found one gene from diptericin and gambicin families. Similarly, we observed three transcripts from lysozyme family with more than 70% identity in the *An. stephensi* SDA-500 and Indian strain. We have generated the phylogenetic tree for each of the family cecropin, defensin, diptericin, gambicin, and lysozyme, respectively (Figures 2A–E). Defensin genes phylogenetic analysis suggested that AMP3 (DEF3) of the *An. stephensi* and *An. gambiae* are different from the main clade. Similarly, in cecropin family AMP6 (CEC3) from *An. gambiae* and *An. stephensi*, AMP7 (gambicin) of *A. aegypti* form a single clade, however, *D. melanogaster* cecropins formed entirely a different clade. Phylogenetic analysis of lysozyme family showed four clades of which one clade is highly specific to *D. melanogaster* (*red color*). As expected *An. stephensi* is more closely related to *An. gambiae, A. aegypti*, and *C. pipiens*, while *D. melanogaster* form a separate clade.

### Constitutive Expression of AMPs Manages Local Infection

**Figure 3:**
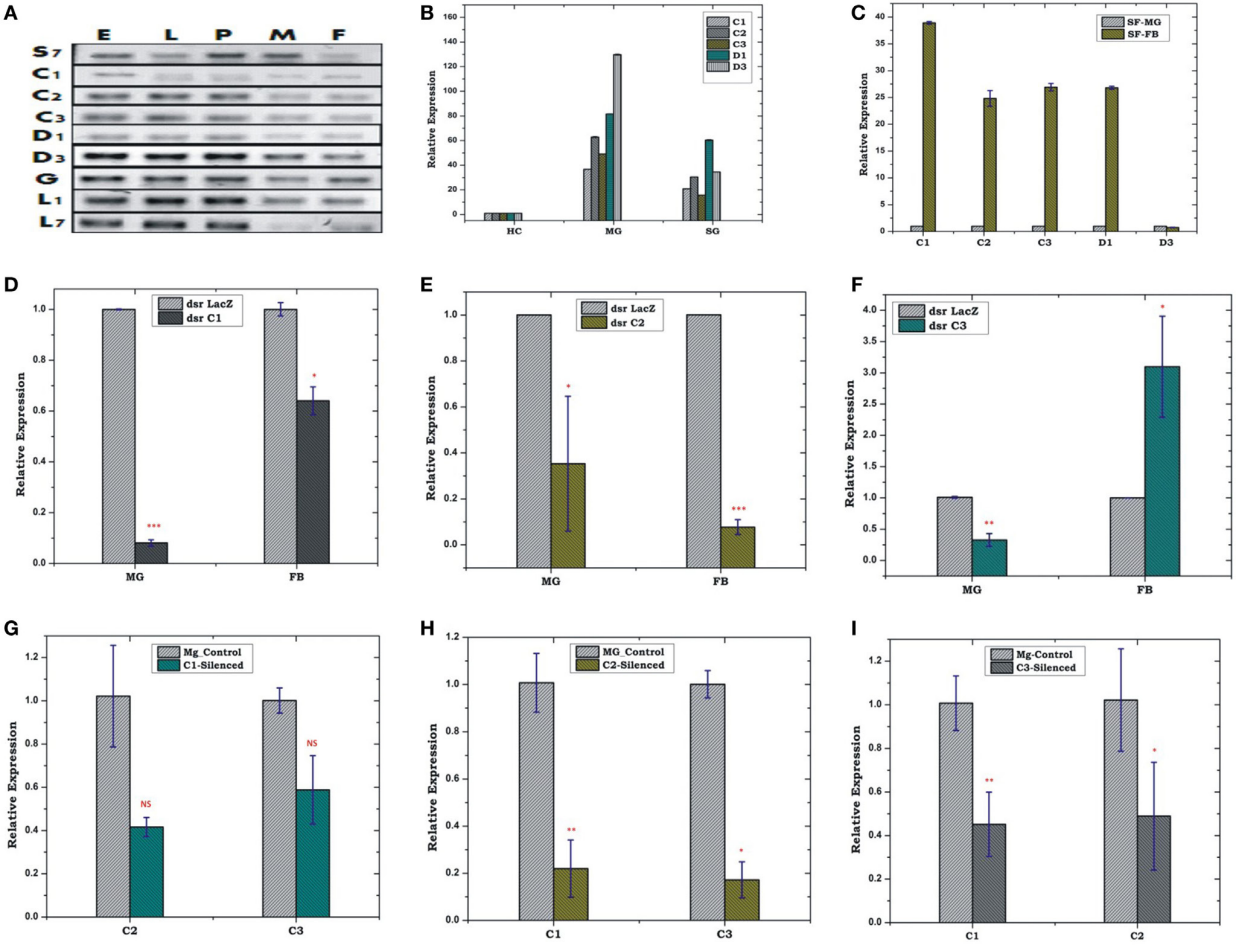
Real time (RT)-PCR expression analysis of antimicrobial peptides (AMPs) during the development of mosquito **(A)**; tissue-specific relative expression analysis **(B,C)**; silencing validation of cecropin family genes **(D–F)**; effect of AMPs silencing in the FB and MG **(G–I)**. The relative expression was monitored using RT-PCR assays. Three independent biological replicates were considered for statistical analysis *viz*. *p < 0.05; **p < 0.005; ***p < 0.0005, using Student’s *t*-test; NS, not significant. Abbreviations: FB, fat body; MG, midgut; SGs, salivary glands.

Constitutive expression of eight antimicrobial peptides defensin (AsD1 and AsD3), cecropin (AsC1, AsC2, and AsC3), gambicin (AsG), and lysozyme (AsLys1 and AsLys7) in RT-PCR analysis, indicated that AMPs significantly contribute to maintain a basal level of immunity throughout the development of the mosquito (Figure 3A). A tissue-specific relative expression analysis of AMPs in the digestive epithelial tissues *viz*. salivary gland (SG), MG, and the HCs, indicated that all the transcripts abundantly expressed in the MG and SGs when compared with HCs (Figure 3B).

### FB-MG Co-ordination and Local Response Management

Our initial observation of elevated AMPs expression in the FB than MG (Figure 3C) confirmed that FB is the principle organ for the production of AMPs, but its co-ordination with HCs and MG, remains largely unknown. Therefore, first, to test whether depletion of AMPs mRNA alters the expression in the FB/MG, we examined relative expression of cecropin family members, 4 days post-dsRNA injection. An effective depletion of all three tested C1, C2, and C3 were observed in the MG. The FB also showed the depletion of AMPs except for C3, which was slightly upregulated (*p* < 0.05) (Figures 3D–F). Next, we tested whether depletion of any one of the AMP member protein alters the expression of the other AMP members of the same family. For example, we observed that effective mRNA silencing of cecropin (C1) family member simultaneously reduces the expression of tested (C2 and C3) cecropin members in the MG (Figures 3G–I).

**Figure 4:**
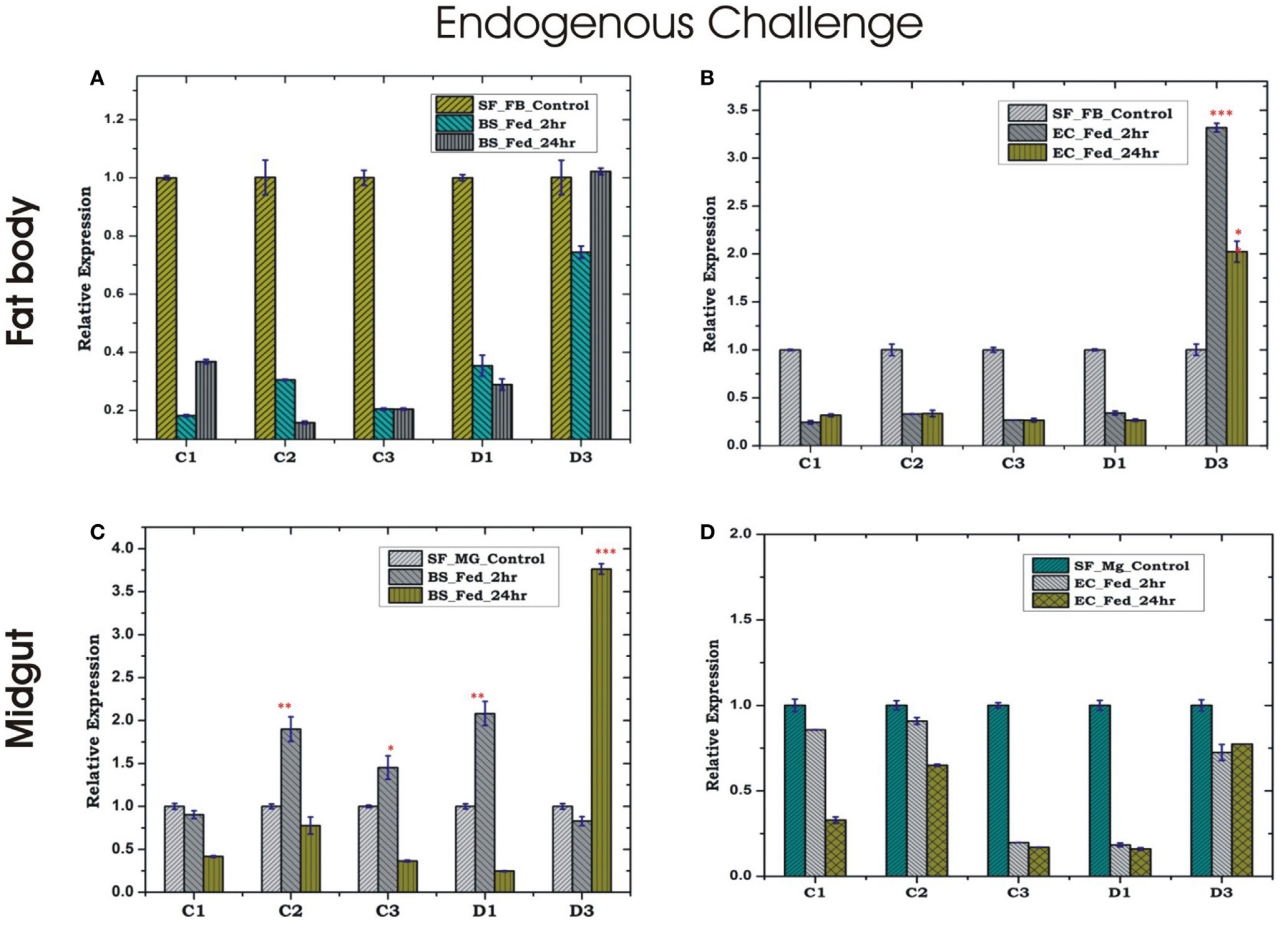
Tissue-specific alteration of antimicrobial peptides expression in response to bacterial feeding/endogenous challenge. Mosquito fat body (FB) response to *Bacillus subtilis* (BS) feeding **(A)**; mosquito FB response to *Escherichia coli* (EC) feeding **(B)**; mosquito midgut (MG) response to BS feeding **(C)**; mosquito MG response to EC feeding **(D)**. *p < 0.05; **p < 0.005; ***p < 0.0005; NS, not significant.

**Figure 5:**
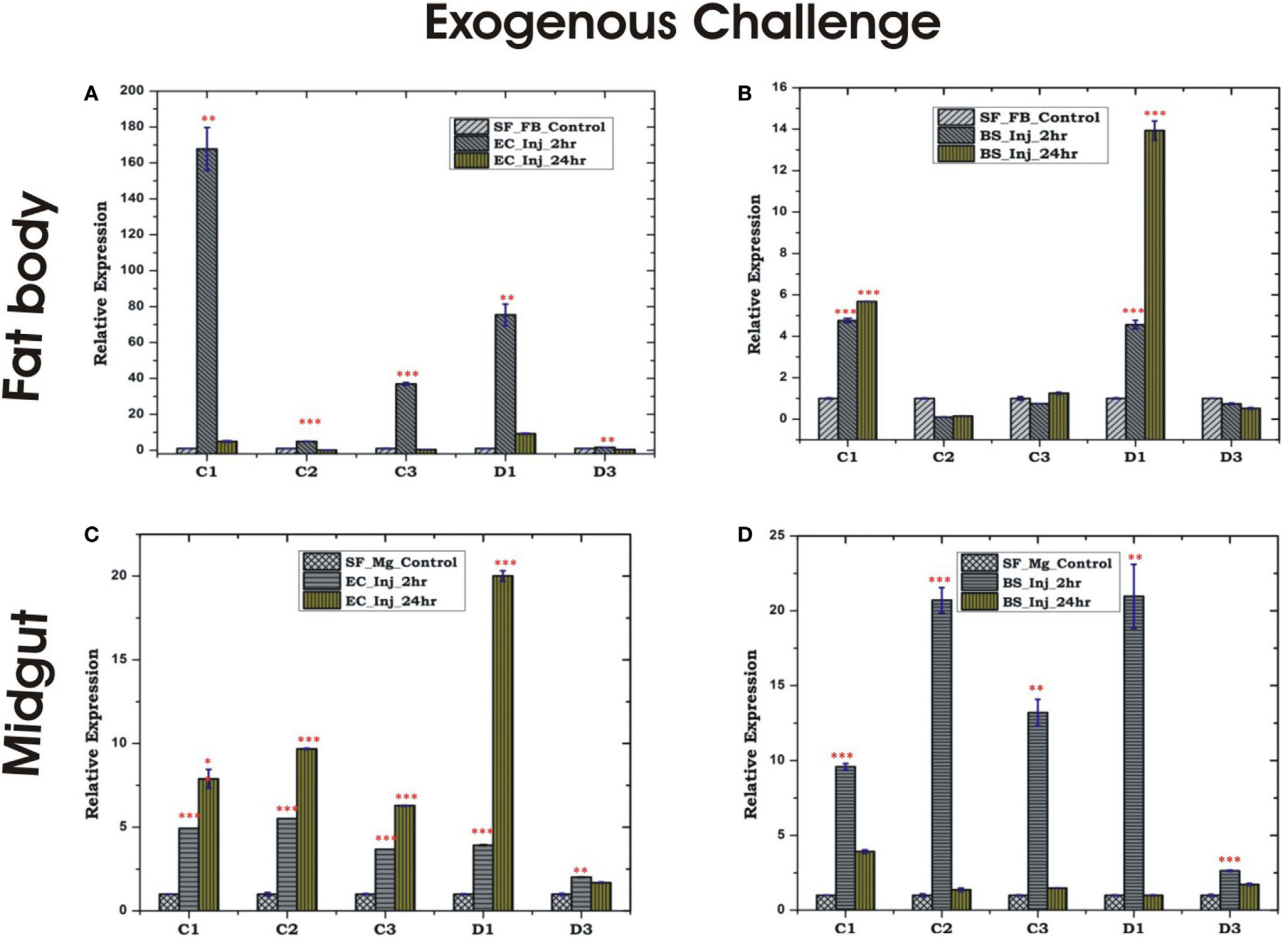
Tissue-specific alteration of antimicrobial peptides expression in response to bacterial injection/exogenous challenge. Mosquito fat body (FB) response to *Escherichia coli* (EC) injection **(A)**; mosquito FB response to *Bacillus subtilis* (BS) injection **(B)**; mosquito midgut (MG) response to EC injection **(C)**; mosquito MG response to BS injection **(D)**. *p < 0.05; **p < 0.005; ***p < 0.0005; NS, not significant.

Endogenous exposure by oral feeding of EC/BS significantly suppresses the basal level expression of AMPs in the FB, except D3 which was upregulated in response to EC feeding (Figures 4A,B). Endogenous exposure of BS resulted in an early upregulation of C2, C3, D1, and late induction of D3 post 24 h of bacterial feeding. However, EC feeding slightly downregulated the expression of all the tested AMPs in the MG (Figures 4C,D). Interestingly, exogenous exposure by microinjection showed significant upregulation of almost all AMPs in the MG as well as the FB, except moderate change of C2/C3 expression in the FB against BS challenge (Figures 5A–D).

### HCs Manage Local and Systemic Immune Responses

**Figure 6:**
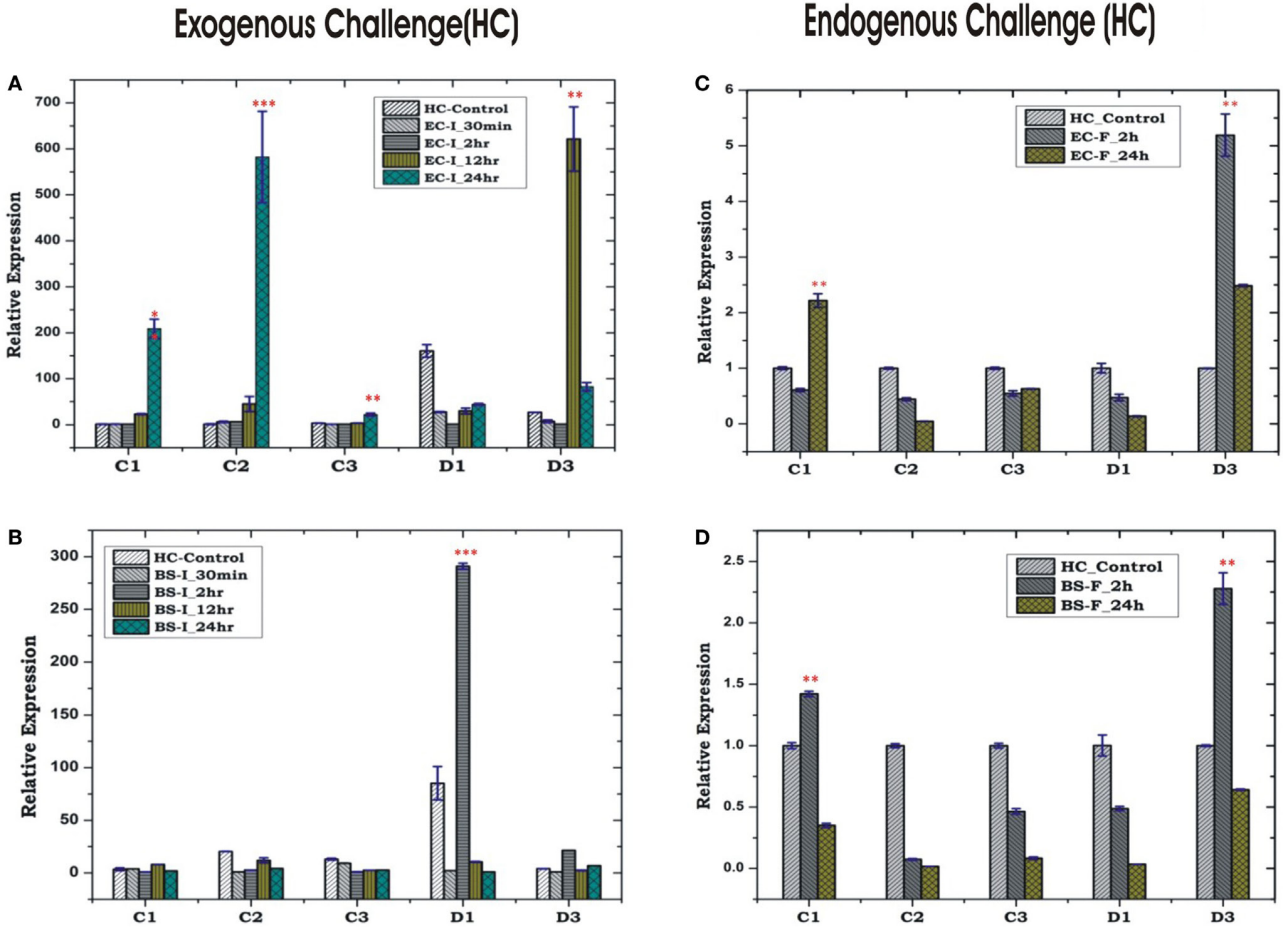
Hemocyte (HC)-mediated antimicrobial peptides response to bacterial challenge (exogenous/endogenous). HC response to *Escherichia coli* (EC) injection **(A)**; *Bacillus subtilis* (BS) injection **(B)**; EC feeding **(C)**; BS feeding **(D)**. *p < 0.05; **p < 0.005; ***p < 0.0005; NS, not significant.

In insects, the systemic immune response is largely managed by HCs, but their co-ordination with local response remains poorly understood, especially in mosquitoes. To clarify this relationship, we first examined the inducible expression of AMPs in the HCs/MG for more elaborated time period, i.e., early (30 min, 2 h), medium (12 h) and late (24 h), against exogenous challenge. A significant upregulation of most of the AMPs was observed at late hours against the microbial challenge in the HCs. As expected most of the AMPs family proteins such as cecropin (C1, C2, and C3) showed significant induction against Gram-negative bacterial (EC) challenge, whereas defensin (D1) was induced against Gram-positive bacterial (BS) challenge (Figures 6A–D). Except mild induction of C1/D3, the endogenous bacterial feeding did not alter AMPs expression in the HCs (Figures 6C,D).

**Figure 7:**
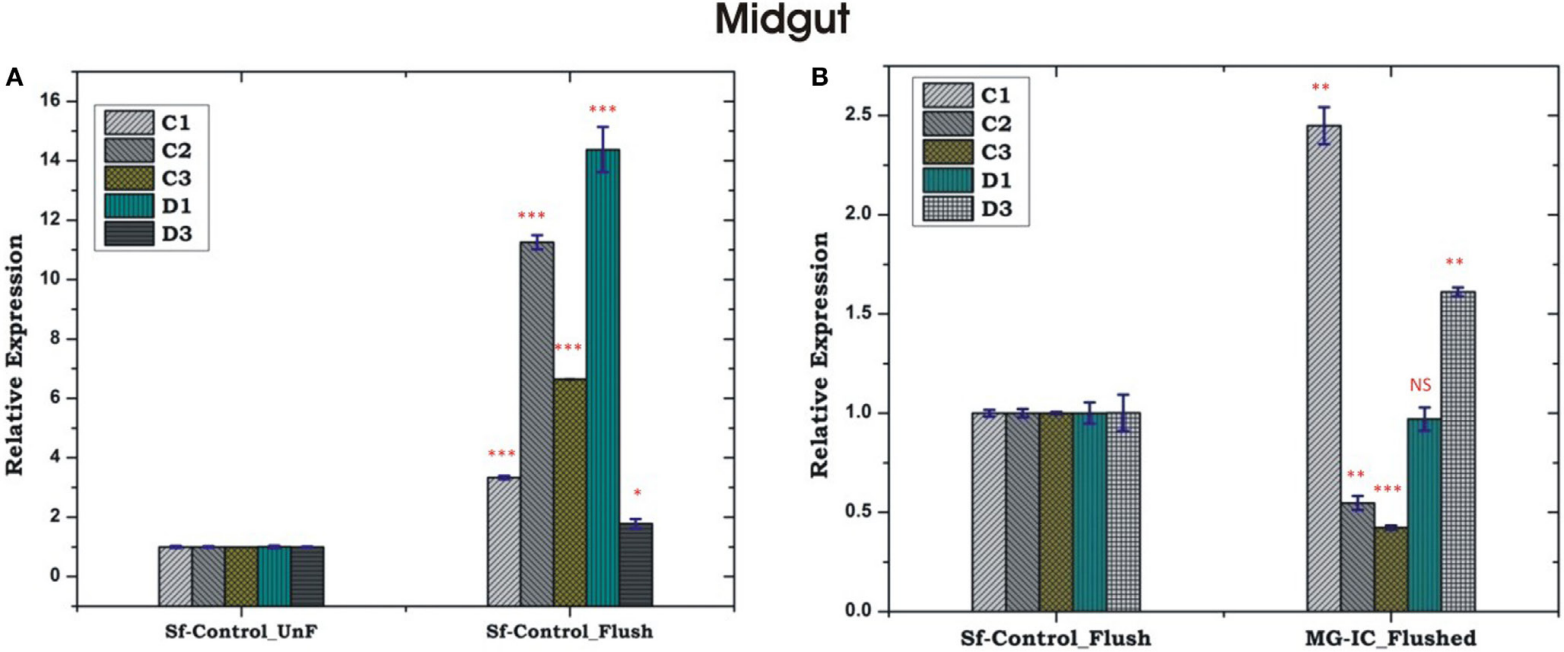
Comparative analysis of antimicrobial peptides in the midgut (MG) collected from hemocyte (HC) flushed and HC un-flushed naïve **(A)**; and immune challenged (IC) mosquitoes **(B)**. *p < 0.05; **p < 0.005; ***p < 0.0005; NS, not significant.

Unlike HCs, MG showed a non-specific induction of AMPs irrespective of the type of bacterial strain, a questionable observation also noted in earlier experiments (Figures 5C,D; Figure S2 in Supplementary Material). Together, these results supported the possibility of physical interactions (attachment/detachment) between HCs and MG during infection. To clarify this complexity, we challenged 3–4 days old naïve adult female mosquitoes with EC or BS. Postmicrobial challenge, first we flushed the HCs and then collected MG from the same mosquito (flushed MG), and monitored AMPs expression. Interestingly, we observed a significant alteration of AMPs expression when compared un-flushed and flushed MG of the naïve as well as exogenous immune challenged mosquitoes (Figures 7A,B).

### Oral Feeding of Bacteria to MG Alters REL/NOS Response in the FB

**Figure 8:**
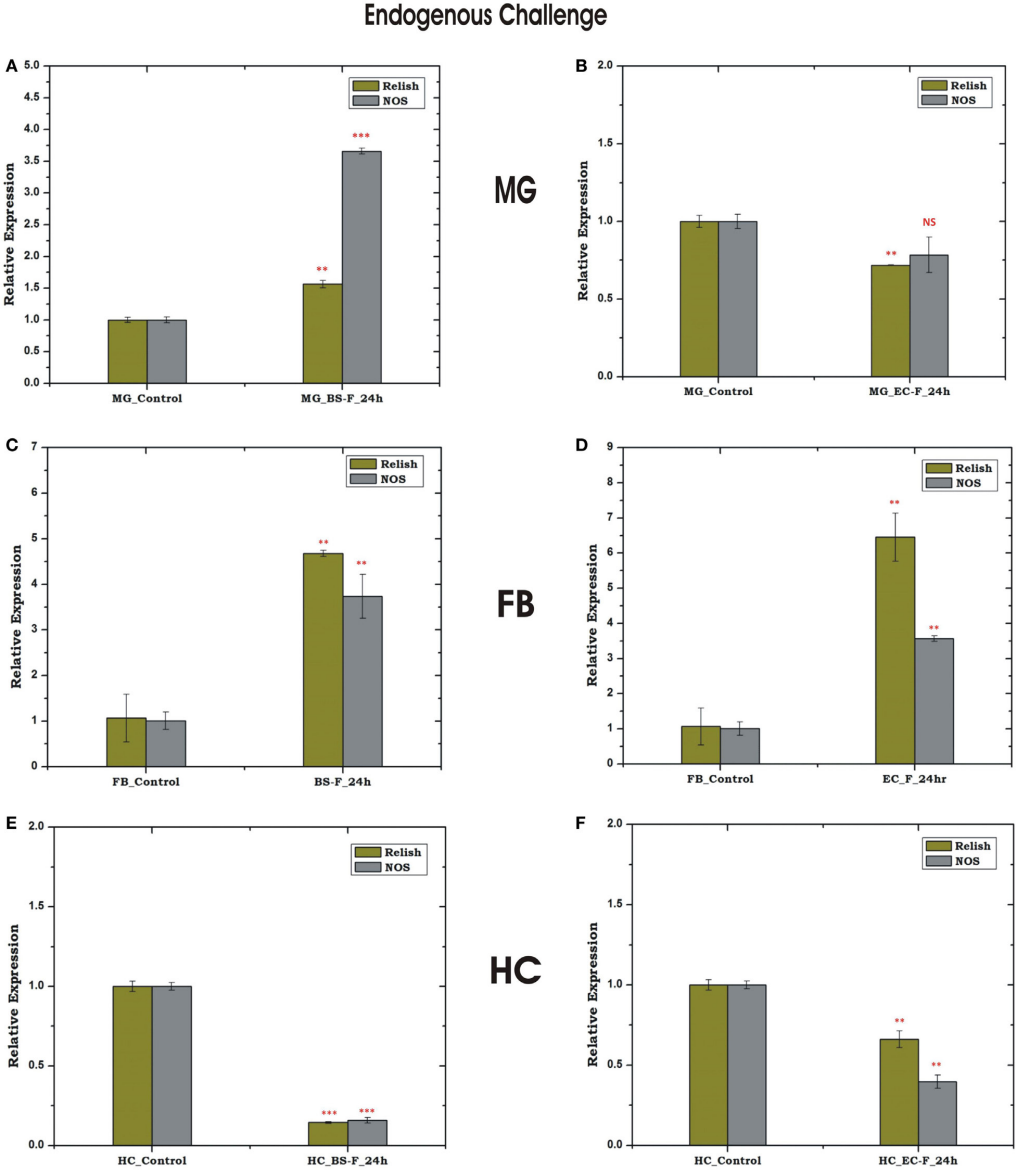
Tissue-specific response of relish (REL) and nitric oxide synthase (NOS) against endogenous (oral feeding) challenge in midgut (MG), fat body (FB), and hemocyte (HC). Mosquito MG response to *Bacillus subtilis* (BS) **(A)**, *Escherichia coli* (EC) **(B)** feeding; FB response to BS **(C)**, EC **(D)** feeding; HC response to BS **(E)**; EC **(F)** feeding. **p < 0.005; ***p < 0.0005; NS, not significant.

*Relish* and *NOS* level were found to be significantly upregulated in the MG and FB, post 24 h of BS feeding (Figures 8A,C), while HCs showed downregulation (Figure 8E). These findings suggested that a direct communication mechanism may exist between MG and FB, which may not require the participation of HC NOS activity (Figures 8A,B). However, unlike BS, the endogenous exposure of EC cause downregulation of REL and NOS in the MG and upregulation in the FB (Figures 8D,F), possibly due to more adaptive nature of Gram-negative bacteria to the mosquito gut.

### Exogenous Microbial Challenges Influence Rel-Mediated HC-FB Immune Communication

**Figure 9:**
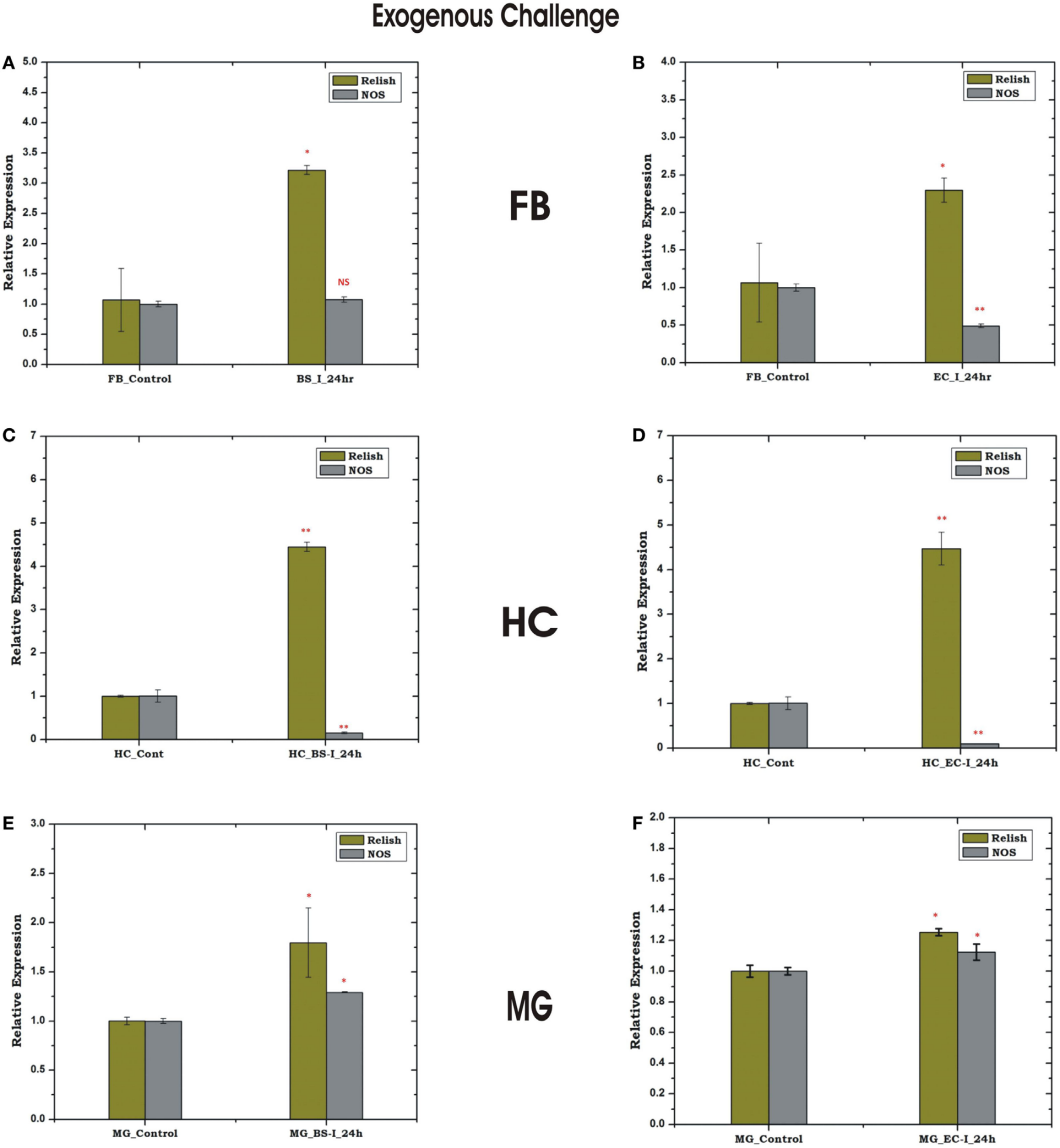
Tissue-specific response of relish and nitric oxide synthase (NOS) against exogenous (thorax injection) challenge in fat body (FB), hemocyte (HC), and midgut (MG). Mosquito FB response to *Bacillus subtilis* (BS) **(A)**, *Escherichia coli* (EC) **(B)** injection; HC response to BS **(C)**, EC **(D)** injection; MG response to BS **(E)**; EC **(F)** injection. *p < 0.05; **p < 0.005; NS, not significant.

To uncover that how FB–HC communicate immune signals, we challenged the naïve mosquitoes with microbial injection and monitored the transcriptional expression of REL and NOS in the FB and HC. Surprisingly, only *Rel* showed a significant modulation irrespective of the nature of microbial injection (Figures 9A–D), suggesting that REL alone could efficiently manage the HC–FB immune network co-ordination and does not require NO participation. However, a mild up regulation of REL and NOS expression was observed in the mid gut, possibly a mixed response elicited by direct interaction of bacteria with the mid gut outer membrane (Figures 9E,F), demanding further experimental verification to establish HC–MG correlation. Thus, to track the possible molecular link we tested the influence of natural gut flora on the HC-MG correlation, as described below.

### Immune Activated HC Limits the Gut Flora Development in the MG

**Figure 10:**
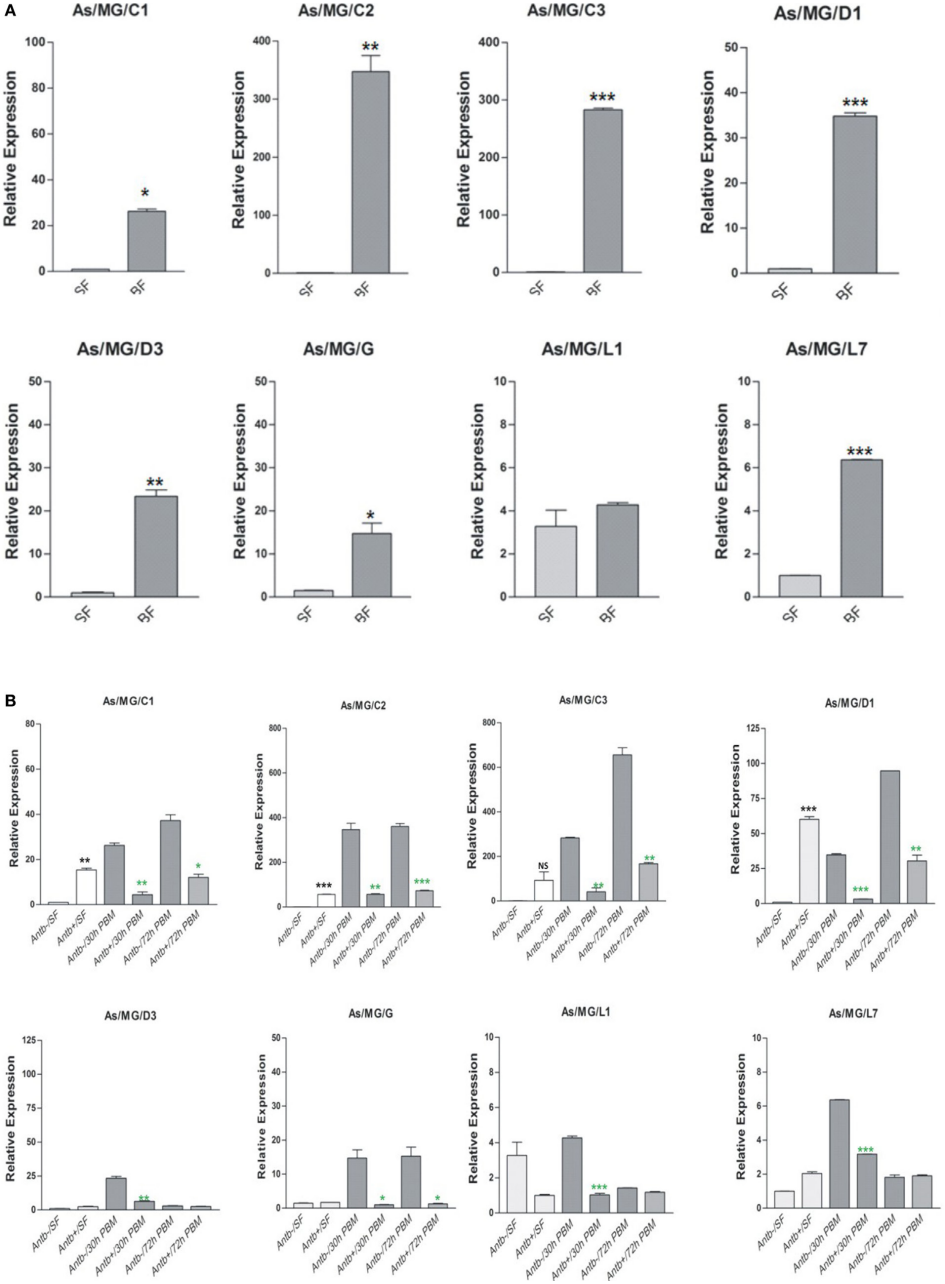
Examination of gut flora influence on antimicrobial peptides (AMPs) expression: effect of blood feeding on AMPs expression in the mosquito midgut (MG) **(A)**; effect of antibiotic treatment on AMPs expression in the mosquito MG **(B)**. *p < 0.05; **p < 0.005; ***p < 0.0005; NS, not significant.

To clarify any immune communication strategies of the MG to HC co-ordination, first we examined the relative expression of AMPs in the MG collected 30 h post blood meal (PBM). Interestingly, in this analysis we observed a significant upregulation of all AMPs in the gut of the blood-fed mosquitoes (Figure 10A). To test the role of proliferated gut flora on the AMP induction, we re-examined and compared the transcriptional response of AMPs in the MG of untreated (*Antb^−^*^ve^) and antibiotic-treated (*Antb*^+ve^) mosquitoes after 30 and 72 h PBM. Interestingly, each family member of AMPs showed a significant downregulation in response to antibiotic treatment at both the time points of PBM (Figure 10B). Unexpectedly, we also observed an intermittent induction of AMPs (e.g., C2/C3/D1) in response to antibiotic treatment in the sugar-fed mosquito MG. Whether this response is transiently required for the maintenance of the physiological integrity of the epithelial tissues or the immune response was elicited by bacterial remnants are yet to be clarified.

**Figure 11:**
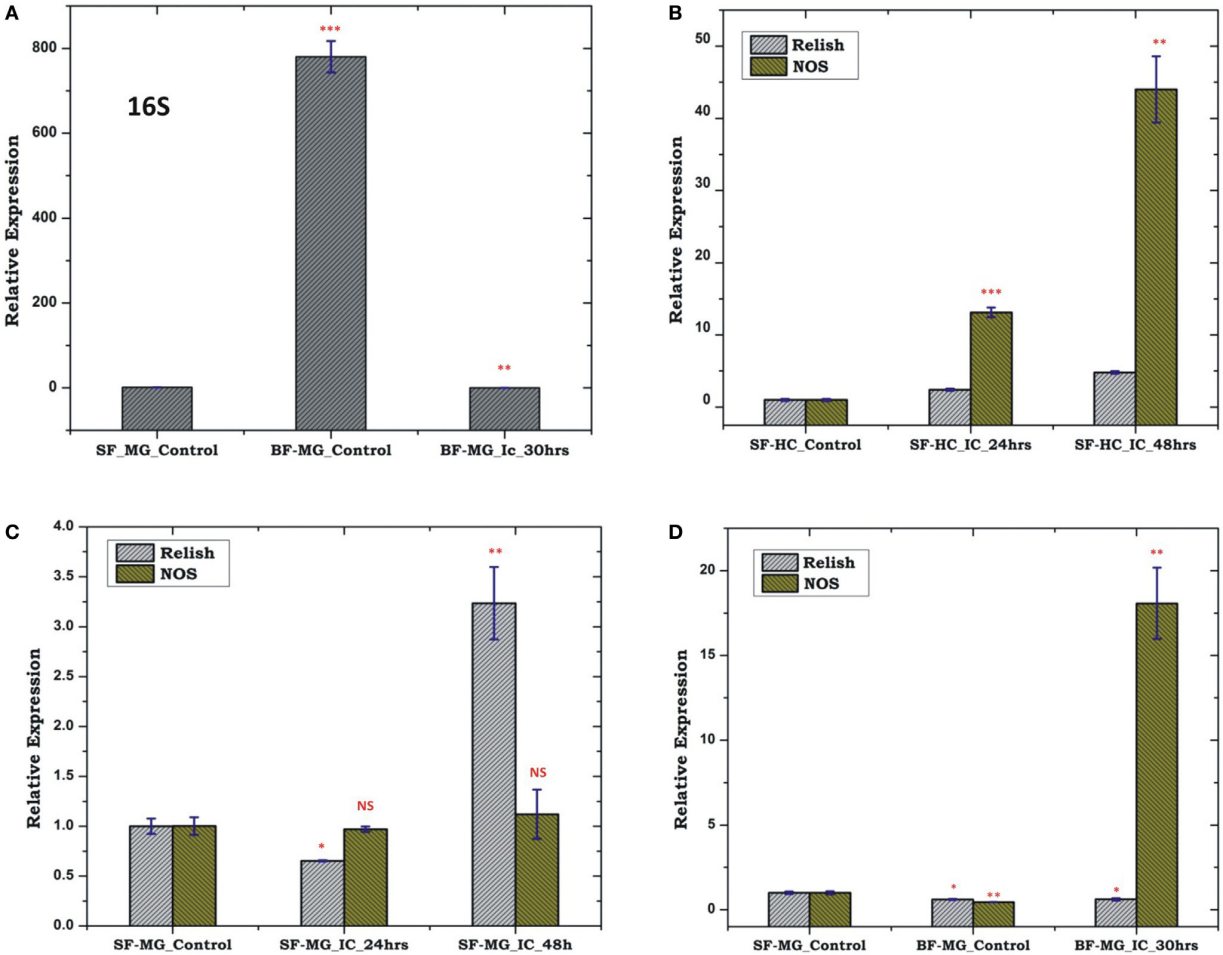
Effect of pre-immunization (thorax injection) and evaluation of relish (REL)/nitric oxide synthase (NOS) expression in the hemocyte (HC)/midgut (MG) during gut flora expansion and blood meal digestion. 16SrRNA based gut flora comparison in the MG **(A)**; REL/NOS expression in the HC in response to pre-immunization **(B)**; REL/NOS expression in response to pre- immunization in the MG of naive sugar-fed mosquito **(C)**; REL/NOS expression in the MG in response to pre-immunization in the blood-fed mosquito **(D)**. *p < 0.05; **p < 0.005; ***p < 0.0005; NS, not significant.

To test whether pre-immunized, i.e., immune activated HC, influences a microbial flora development, we pre-immunized 3–4-day old adult female mosquitoes with a mixed paste of live Gram-positive/Gram-negative bacteria and kept for 24 h before blood meal. In our comparative analysis, we did not observe any significant change in the AMPs expression in the pre-immunized blood-fed mosquito guts (Figure S4 in Supplementary Material), but noticed a significant reduction of the bacterial load as measured by 16S rRNA expression (Figure 11). Although such direct evidence of HC-gut relation is not yet established, our data suggested that immune activated HCs may have cross tissue regulation ability over gut flora proliferation. In these experiments, we further observed that a consistent upregulation of NOS than REL in the HCs up to 48 h post challenge in the non-blood-fed mosquitoes (Figure 11B). In case of the MG, the exogenous challenge with mixed bacterial exposure did not alter the NOS/REL expression significantly, except slight upregulation of REL post 48 h of challenge (Figure 11C). Interestingly, in contrast to very limited change for REL/NOS in HCs (Figure S5 in Supplementary Material), we observed an exclusive rapid induction of NOS in the blood-fed MG of the pre-immunized mosquitoes (Figure 11D). Taken together, we hypothesize that NOS and REL may have a synergestic role in limiting the gut flora expansion as well as HC-mediated immune responses.

## Discussion

Even though the knowledge on cross tissue communication is limited, our initial data confirms that AMPs play an important role to fight local infections. Data also indicated that HCs relatively do not express AMPs locally, until they are signaled or exposed to any antigen. With our initial experiments with dsRNA-mediated gene silencing, we hypothesize that FB and MG may carry the ability to manage the fine adjustment of the AMPs requirement during any local infection. To test and verify this correlation, next, we independently examined and compared the differential expression of selected AMPs in the FB and MG against endogenous and exogenous exposure of BS (Gram-positive) and EC (Gram-negative) bacteria.

Interestingly, both FB and MG caused mild suppression of AMPs expression, except to a significant upregulation in response to BS oral feeding, indirectly suggesting that mosquitoes gut environment may favor a cost-effective immune tolerance against symbiotically associated Gram-negative bacteria (41, 42), than a non-adaptive member of virulent bacterial family members, especially BS which also releases toxic proteins (43). In contrast to the above, a significant upregulation of almost all AMPs in the FB as well as MG in response to the exogenous exposure of both EC/BS, suggested that FB has an ability of fine adjustment of AMPs expression to meet and supply the AMPs on-demand basis (44). However, striking upregulation of AMPs in the mosquito MG remains a questionable observation, by the fact that FB and MG do not come in direct contact at any stage of the infection (26, 45).

Emerging evidence strongly suggests that mosquito HCs are the key partners of systemic immune responses (7, 39, 46–48), but their immune relation with other organ(s) remains unclear. When given an exogenous challenge, our experimental data indicated that HCs are not only specialized to discriminate antigen but a consistent upregulation of AMPs till late hours, suggested their important role to clear off the remaining persistent bacteria in the hemolymph, a mechanism proposed in insects (49). We hypothesized that depending on the nature of infection the HC immune response may be different than other tissues because (i) AMPs do not naively express in HC but are late inducible; (ii) HC may have dual ability to face injury responses to minimize the tissue damage during early hours (28, 50). Furthermore, it may also be critical to access the HC–FB correlation, specifically due to the complexity associated with free circulating HCs than fixed but loosely distributed FB tissue, where bacteria may encounter to FB/HC during circulation within hemolymph. Therefore, we tested whether circulating mosquito blood cells, i.e., HCs play any role in this interorgan communication, i.e., FB/MG immune network management. A comparison between un-flushed and flushed MG indicated that HC attachment/detachment may account for a mixed response in the MG (Figures 7A,B). The possible reason of these observations may be due to exogenous exposure of specific microbes in the thorax, which may encounter with the outer lining of the MG epithelia, i.e., basal lamina, eliciting a mixed immune response at the interface of HCs–MG attachment.

In the mosquito *An. gambiae*, it has been demonstrated that ~25% HCs are sessile in nature and dominantly associated with abdomen (51). In fact, any exogenous or endogenous microbial challenge significantly alters systemic and local immune responses, where HCs may play interorgan communication between MG and FB, probably through immune signal activation and MG attachment during infection. Though it is unclear that whether sessile HCs also contribute toward “systemic-cum-local” responses, however, we interpreted that a synergistic relationship of early induction of AMPs in FB, and late induction in HC may not only manage the systemic infection but also establish a successful co-ordination with local response through immune signaling mechanism (52).

Nitric oxide, a by-product of NOS activity, serve as an important immune signaling modulator in insects (53). In *Drosophila* NO induces innate immune genes upon natural infection of Gram-negative bacterial infection (26). While in the *Anopheline* mosquitoes, transcriptional upregulation of NOS not only kill *Plasmodium* in the gut (15, 17, 20–23, 54), but also regulate HC-mediated bacterial killing during systemic infection with EC (24). IMD signaling pathways regulate the expression of several innate immune genes during microbial challenge *via* activation of transcription factor *Rel* (55–57).

Thus, to trace the possible molecular link associated with the interorgan flow of signals, we selectively profiled NOS as well as REL expression during exogenous as well as endogenous exposure. A significant alteration in the level of *Rel* and *NOS* expression, i.e., upregulation in the MG and FB, while downregulation in the HC, suggested that a direct communication mechanism may exist between MG and FB, requiring a negligible level of HC NOS activity. Alternatively, the upregulation of *REL* and *NOS* in the FB can also be interpreted by the generation of intestinal oxidative stress after bacterial feeding (58). This elevation of the ROS activity was determined by the DCFDA staining of the endogenous challenged MG (Figure S3 in Supplementary Material). The resulting ROS may send a putative signal to the FB which may result in the NOS upregulation (24). Furthermore, we observed that oral supplement of EC does not influence Rel/NOS expression in the gut than the FB. Since, enterobacteria such as EC are thought to constitute the pre-dominant gut flora of adult female mosquitoes (42, 59), we interpret that endogenous exposure of EC may not be immunogenic as BS. But EC may be more invasive than *Bacillus* which may disrupt the epithelial barrier of the mid gut, eliciting the FB and HC response (60). Taken together, our data summarize that MG to FB communication may largely depend on REL while MG to HC through NO signaling. On the other hand, our data also suggested that FB to HC is dominantly managed by the immune network activated REL alone in the adult mosquitoes, but it remains a challenge to uncover MG to HC signaling path.

Previous studies demonstrate that blood meal induces several-fold increase in the population of gut flora, which may also influence the immune response of the mosquito (25, 41, 61). To clarify MG -HC immune communication strategy, we tested a hypothesis whether (i) the blood meal-induced expansion of natural gut flora influences the REL/NOS expression in the HC or (ii) pre-immune activated HC influences this gut flora expansion. Our findings suggested that a synergistic actions of AMPs enables optimal regulation of the native microbial gut flora proliferation during blood meal uptake and digestion (62). Though, a transient induction of AMPs could also be expected in early hours in response to temperature switch from vertebrate blood (37°C) to a 28°C gut temperature of naïve mosquito (63). However, we believe late induction of AMPs after 30 h of blood feeding may dominantly depend on the proliferation of naïve bacteria as reported earlier (64, 65). Next, our data also suggested that immune activated HCs may have the cross tissue regulation ability, over gut flora proliferation, possibly through NOS, because we observed a consistent upregulation of NOS than REL in the HCs up to 48 h post challenge in the non-blood-fed mosquitoes (Figure 11B). Surprisingly, a rapid induction of NOS in the blood-fed MG of the pre-immunized mosquitoes allowed us to hypothesize that NOS and REL may have synergetic role in limiting the gut flora expansion as well as HC-mediated immune responses.

## Conclusion

**Figure 12:**
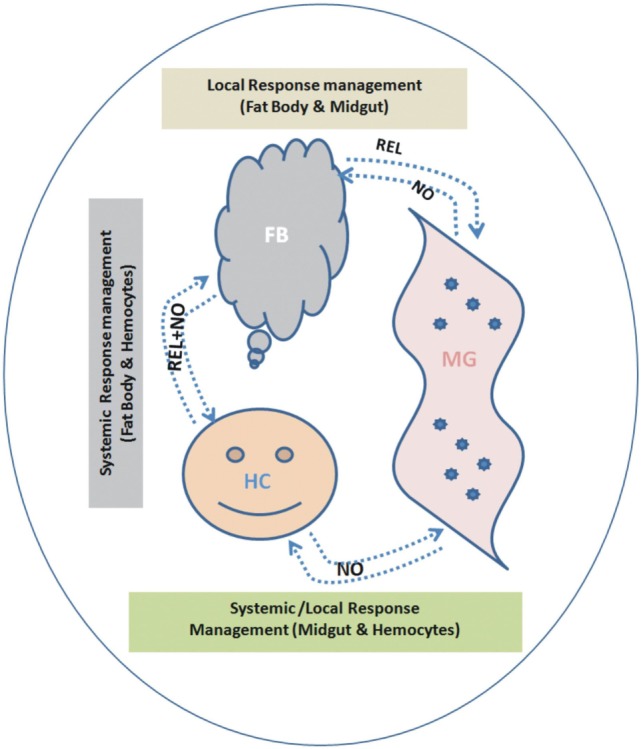
Proposed working hypothesis/model for future validation and establishment of a possible co-ordination and participation of relish (REL)/nitric oxide (NO) synthase controlling antimicrobial peptides response. To establish possible flow of signal relationship, we interpreted the cross tissue experimental data as below: (i) *Endogenous bacterial feeding*: demonstrating NO dominantly regulate flow signal from midgut (MG)-to-FB and/or MG-to-hemocyte (HC) (Table S2 in Supplementary Material). (ii) *Exogenous bacterial injection*: demonstrating REL dominantly regulate flow signal from FB-to-MG; while NO regulate HC-to-MG (Table S2 in Supplementary Material). A synergistic relationship of REL/NO between FB and HC was established using combined data from individual as well mixed bacterial challenge experiments.

To understand the interorgan immune communication strategy, in the present investigation, we not only examined the AMPs expression in three important mosquito tissues such as MG, FB, and HCs, but also traced the molecular link of the signaling transmitters, i.e., REL and the synthesizer of NO, i.e., NOS, controlling AMPs expression. We found that each tissue has unique ability to respond local/systemic challenges; however, HCs are more specialized than the gut and FB to recognize and discriminate-specific antigens. This study also demonstrates that both REL and NO participate in the overall management of the interorgan immune communication, but each tissue specifically maintains the interorgan flow of signals (Figure 12).

## Author Contributions

TDD, PS, RD, and KCP conceived and designed the experiments. TDD, PS, TT, DS, ST, SK, JR, CC, RK, and VS performed the experiments. TDD, PS, TT, RD, and KCP analyzed the data. RD and KCP contributed reagents/materials/analysis tools. RD, TDD, PS, and KCP wrote the paper. All authors read and approved the final manuscript.

## Conflict of Interest Statement

The authors declare that the research was conducted in the absence of any commercial or financial relationships that could be construed as a potential conflict of interest.
