# Supplementary material for: Interorgan Molecular Communication Strategies of “Local” and “Systemic” Innate Immune Responses in Mosquito Anopheles stephensi
Source: Front Immunol. 2018 Feb 20;9:148. doi: 10.3389/fimmu.2018.00148 (PMC5826171; doi:10.3389/fimmu.2018.00148)
Supplement: Supplementary file 1 [file Data_Sheet_1.PDF]

Supplemental Data

Interorgan molecular communication strategies of 'local' and 'systemic' innate immune responses in mosquito *Anopheles stephensi*

Tanwee Das De[$], Punita Sharma[$], Tina Thomas, Deepak Singla, Sanjay Tevatiya, Seena Kumari, Charu Rawal, Jyoti Rani, Vartika Srivastva, Ramandeep Kaur, Kailash C. Pandey, Rajnikant Dixit[*]

Host-Parasite Interaction Biology Group, ICMR-National Institute of Malaria Research, Sector-8, Dwarka, Delhi-110077 (India)

[*] Correspondance

Phone : 91-11-25307217; Fax: 91-11-25307160

E-mail: dixit2k@yahoo.com

[$]Contributed equally

Supplemental Table 1: Identification of AMPs and Lysozymes in *An. stephensi* SDA-500 genome

ID	Type	Family	E-value	%identity	Cov.	Size (DNA/AA's)		Best Hit
						An. stephensi	*An. gambiae*	
**ASTE007107-RA**	AMP4	Cecropin (CEC1)	3E-049	87.43	94	177/58	177/58	Agam:AMP4
**ASTE007106-RA**	AMP5	Cecropin (CEC2)	8E-035	81.56	99	180/59	177/58	Agam:AMP5
**ASTE007108-RA**	AMP6	Cecropin (CEC3)	4E-028	87.62	57	189/62	183/60	Agam:AMP6
**ASTE010339-RA**	AMP7	Cecropin (CEC4)	5E-042	82.18	98	4496/64	204/67	Agam:AMP7
**ASTE011281-RA**	AMP1	Defensin (DEF1)	5E-049	85.71	83	291/96	309/102	Agam:AMP1
**ASTE002821-RA**	AMP2	Defensin (DEF2)	3E-025	80.41	61	2100/699	243/80	Agam:AMP2
**ASTE000973-RA**	AMP3	Defensin (DEF3)	3E-024	78.29	84	195/64	204/67	Agam:AMP3
**ASTE011101-RA**	AMP10	Defensin (DEF4)	3E-036	87.12	46	593/90	285/94	Agam:AMP10
**ASTE000972-RA**	AMP11	Defensin (DEF5)	0.000002	77.08	45	189/62	207/68	Agam:AMP11
**ASTE009529-RA**	AMP8	Diptericin (DPT)	1E-079	89.7	70	675/109	333/110	Agam:AMP8
**ASTE002252-RA**	AMP9	Gambicin (GAM1)	7E-067	85.08	89	462/81	279/81	Agam:AMP9
**ASTE005846-RA**	LYS1	Lysosome (LYSC1)	3E-087	80.83	97	927/308	423/140	Agam:LYS1
**ASTE005846-RA**	LYS2	Lysosome (LYSC2)	2E-029	75.26	92	927/308	423/140	Agam:LYS2
**ASTE007041-RA**	LYS6	Lysosome (LYSC-6)	0	81.24	93	2841/946	2544/847	Agam:LYS6
**ASTE005878-RA**	LYS7	Lysosome (LYSC-7)	7E-114	83.94	93	1463/298	462/153	Agam:LYS7

Fig. S1

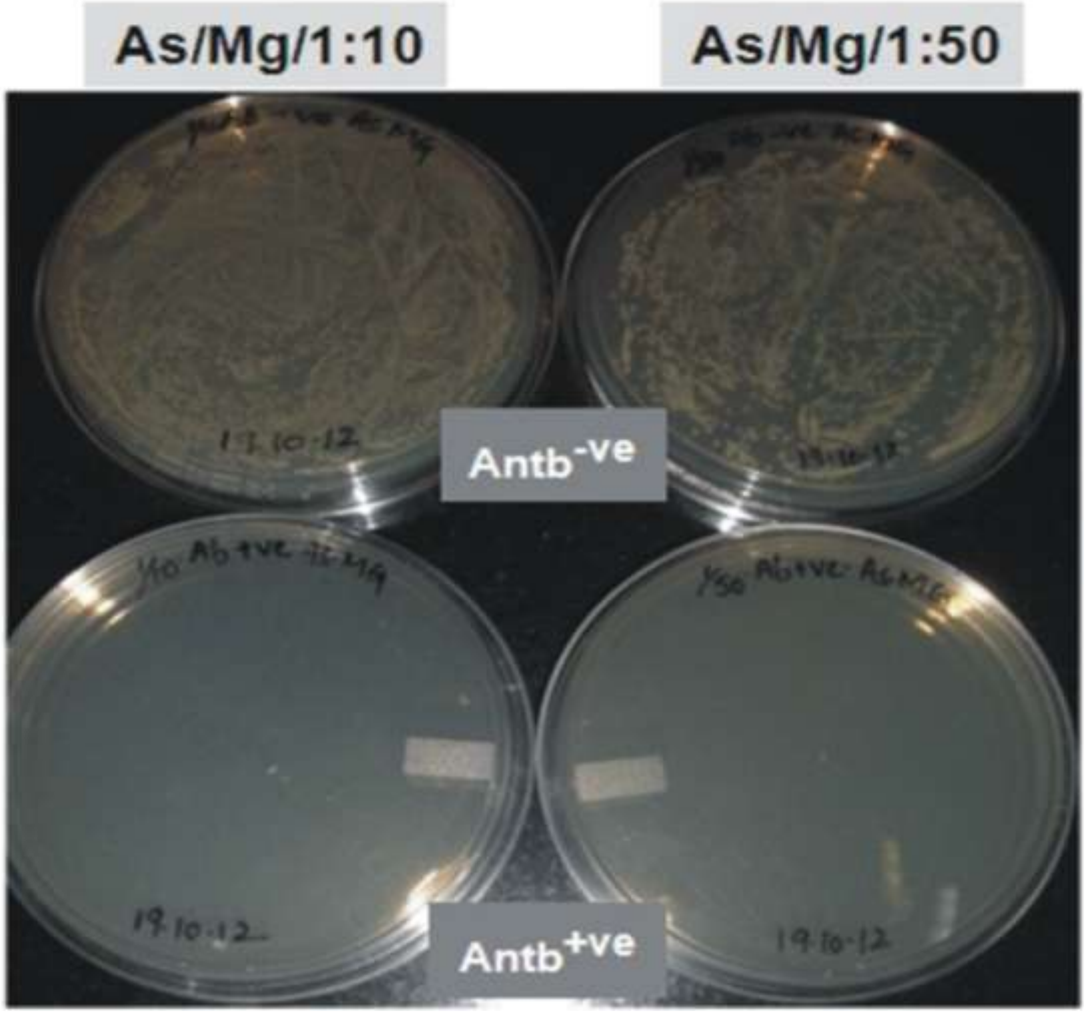

Fig S1: Examination of gut Flora removal: Gut flora removal after antibiotic treatment was monitored by plating the homogenized midgut (Mg) tissue on LB-agar plate at 1:10 and 1:50 dilutions.

Fig.S2

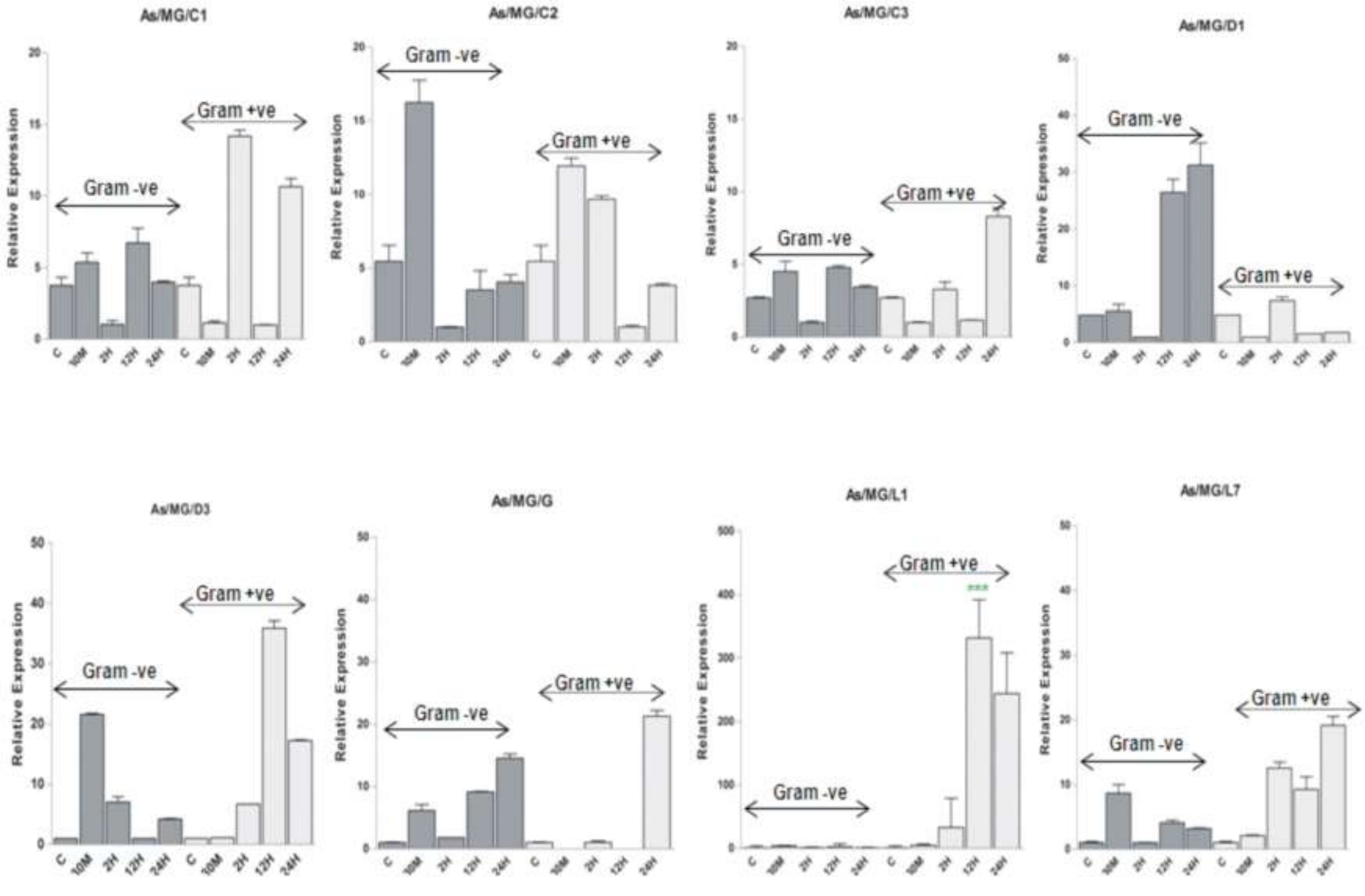

Fig S2: Midgut (MG) AMP expression analysis in response to exogenous challenge with detail time course viz. early (30 min+ 2hr) medium (12 hr) & Late (24hr)

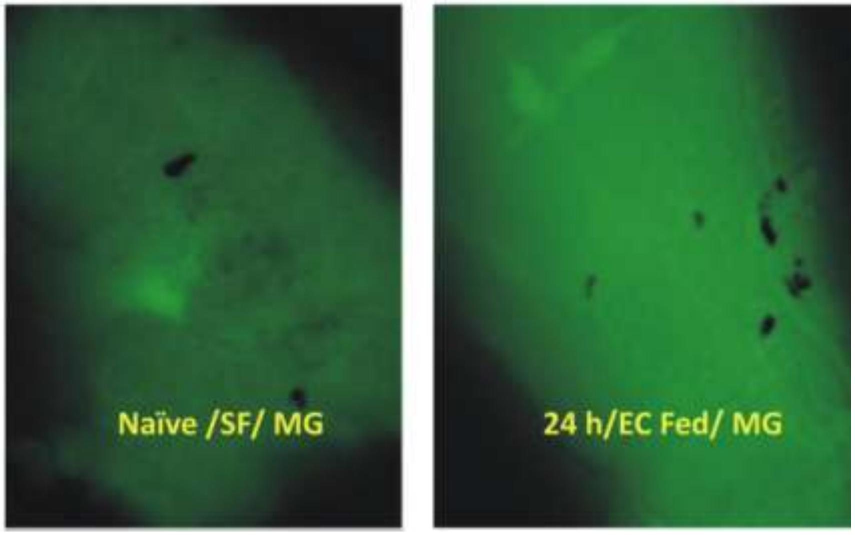

Fig. S3 DCFDA staining assay demonstrating increased ROS production in the gut of E.coli fed mosquitoes**.**

Fig. S4

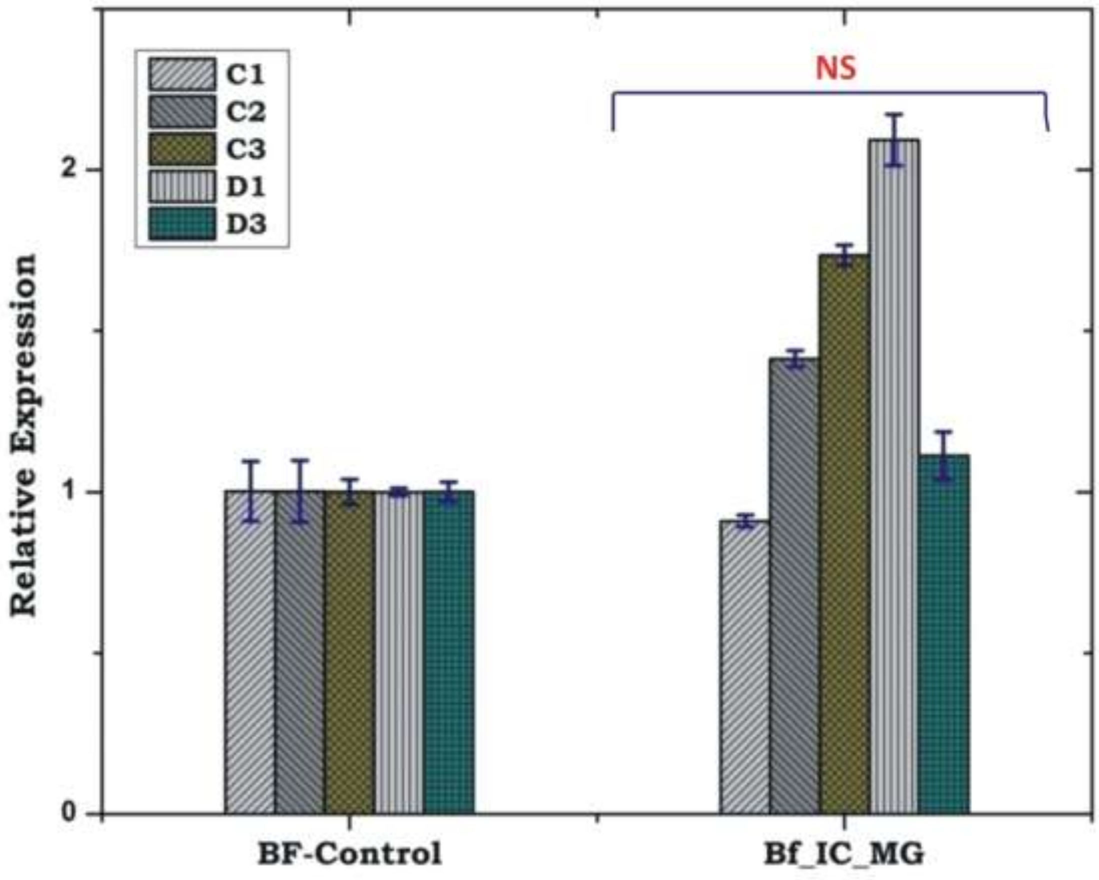

Fig S4: Effect of pre-immunization on blood fed induced AMP expression in midgut.

Fig. S5

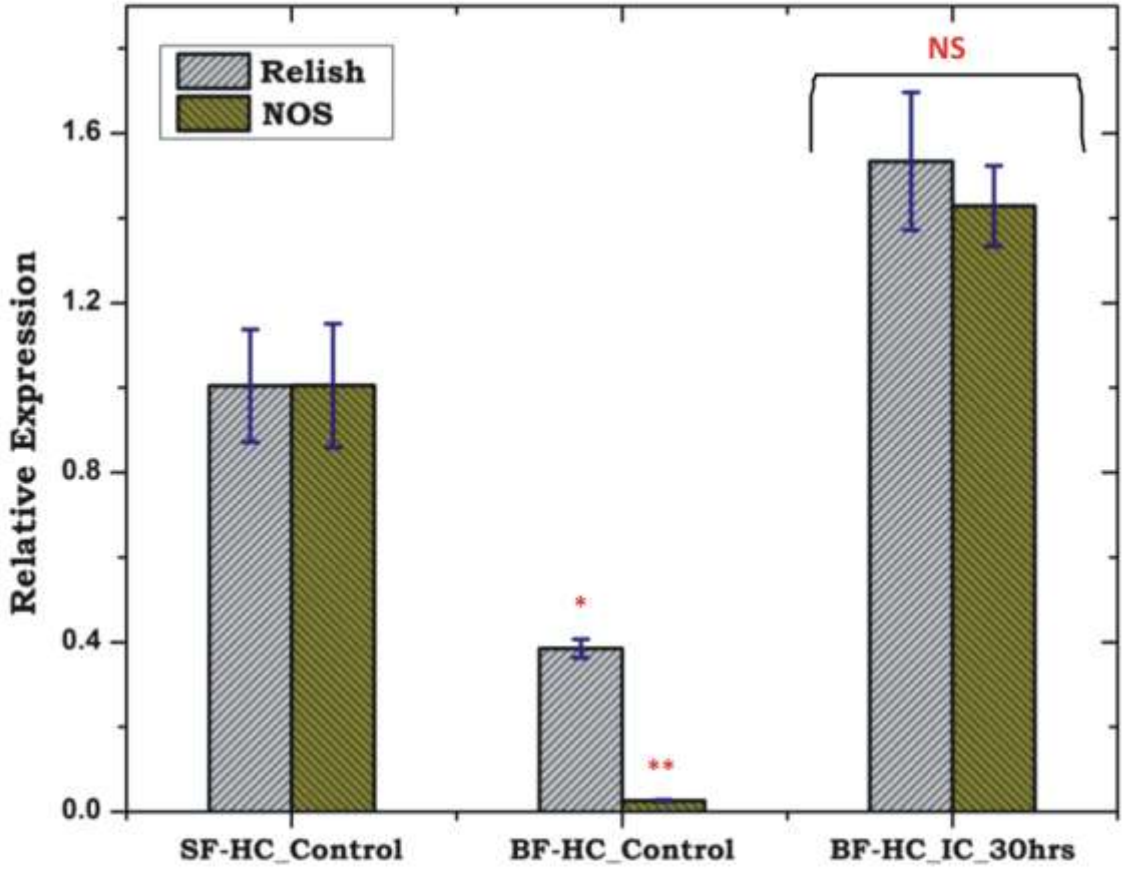

Fig S5: Expression profile of REL/NOS in hemocyte (HC) in response to blood feeding and pre-immune challenge.

Supplemental Table 2: Tissue specific experimental data interpretation to understand the general response of REL/NOS under distinct exposures

<u>Tissue</u>	<u>Exogenous Infection</u>	<u>NOS/REL Response</u>	<u>Endogenous Injection</u>	<u>Interpreted General Response</u>
Fat Body	*E.coli* Injection	REL-Up-Regulation<br>NOS-No-Change	*E.coli* Feeding	NOS+REL-Up-Regulation
	B. subtilis Injection	REL-Up-Regulation<br>NOS-No-Change	*B.subtilis* Feeding	NOS+REL-Up-Regulation
Hemocyte	*E.coli* Injection	REL-Up-Regulation<br>NOS- Down-Regulation	*E.coli* Feeding	REL/NOS- No Change
	B.subtilis Injection	REL-Up-Regulation<br>NOS-Mild Suppression/No-Change	*B.subtilis* Feeding	NOS+REL-Mild Suppression
Midgut	*E.coli* Injection	REL/NOS-No change	*E.coli* Feeding	NOS/REL- No-Change
	B.subtilis Injection	REL/-Mild -Up-Regulation<br>NOS/No-Change	*B.subtilis* Feeding	NOS-Up regulation<br>REL-No Change

Supplemental Table 3: List of AMPs Primers

Name	Primer Sequence	Size (bp)
**As_Cec1_F**	AACCAACCGAACCGTATCAA	160
**As_Cec1_R**	TTTCTCAGCTGCCTTGAACA	
**As_Cec2_F**	AAGCTGCTCTTTCTCGTTGC	183
**As_Cec2_R**	GTGAGGTACGCCCTATCCAA	
**As_Cec3_F**	ATGCGCAGAGGAACTCACTT	160
**As_Cec3_R**	ACCGAAACCAGAACAAATCG	
**As_Def1_F**	GATGAACTGCCCGAAGAGAC	159
**As_Def1_R**	TTGCTGGCTGTTGCAGTATC	
**As_Def3_F**	GCGACCGTGTGTAGTTCAGA	233
**As_Def3_R**	CACTCATCCTGGTCGCTACA	

**As_Gamb_F**	ACTGTGGCTACGGGTACGTC	168
**As_Gamb_R**	GCTTGTTCTTCCGGTGTGAT	
**As_Lys1_F**	ACGCTTGGTATGGATGGAAG	207
**As_Lys1_R**	TCGACATGCTTTGTTTCAGC	
**As_Lys7_F**	GTACCACGGACACTGGGTCT	222
**As_Lys7_R**	TGTTGGATCATTACGGAGCA	
